# Oral Administration of Polyethylene Microplastics Induces BPA-Associated Antioxidant Activation and Synaptic-Related Transcriptional Responses in the Rat Prefrontal Cortex

**DOI:** 10.3390/nu18121892

**Published:** 2026-06-11

**Authors:** Maria del Mar Ribas-Taberner, Maria Magdalena Quetglas-Llabrés, Llucia García-Moll, Manuel Jiménez-García, Joan Truyols-Vives, Silvia Tejada, Miguel D. Ferrer, Manuel Miró, Antoni Sureda

**Affiliations:** 1 Research Group on Community Nutrition & Oxidative Stress, Department of Fundamental Biology and Health Sciences, University of the Balearic Islands, E-07122 Palma, Illes Balears, Spain; m.ribas@uib.cat (M.d.M.R.-T.); m.quetglas@uib.cat (M.M.Q.-L.); 2 Health Research Institute of Balearic Islands (IdISBa), E-07120 Palma, Illes Balears, Spain; manuel.jimenez@uib.cat (M.J.-G.); silvia.tejada@uib.es (S.T.); miguel-david.ferrer@uib.es (M.D.F.); manuel.miro@uib.es (M.M.); 3 CIBERobn (Physiopathology of Obesity and Nutrition), Instituto de Salud Carlos III, E-28029 Madrid, Madrid, Spain; 4 Flow Injection and Trace Analysis Group (FI-TRACE), Department of Chemistry, University of the Balearic Islands, E-07122 Palma, Illes Balears, Spain; llucia.garcia@uib.cat; 5 Research Group on Neurophysiology, Department of Biology, University of the Balearic Islands, E-07122 Palma, Illes Balears, Spain; 6 Molecular Biology, Health Geography, and One Health Research Group (MolONE), Department of Fundamental Biology and Health Sciences, University of the Balearic Islands, E-07122 Palma, Illes Balears, Spain; joan.truyols@uib.cat; 7 Renal Lithiasis and Pathological Calcification Group (LiRCaP), Research Institute of Health Sciences (IUNICS), University of the Balearic Islands, E-07122 Palma, Illes Balears, Spain

**Keywords:** microplastics, bisphenol A, prefrontal cortex, oxidative stress, inflammation, neurotoxicity, acute exposure

## Abstract

**Background/Objectives**: The pervasive presence of microplastics (MPs) and plastic-associated chemicals has raised concerns regarding their potential effects on the central nervous system. Polyethylene (PE), widely used in food-contact materials, can carry bisphenol A (BPA), an endocrine disruptor with oxidative and neuroactive properties. Although both MPs and BPA can cross biological barriers, their acute effects on the prefrontal cortex (PFC) remain poorly understood. The aim of the study was to evaluate the acute impact of orally administered free BPA, free MPs, and BPA adsorbed onto PE MPs (PE–BPA) on oxidative stress, inflammation, and gene expression in the PFC of Wistar rats. Animals received a single dose of BPA, PE–BPA, PE alone, or vehicle. **Methods**: Biochemical and transcriptional analyses were performed to evaluate the antioxidant and inflammatory responses as well as the potential changes in synaptic-related gene expression. **Results**: BPA-containing treatments produced selective early molecular responses. Catalase (CAT) and glutathione S-transferase (GST) activities were significantly increased in the PE–BPA group, with GST being also elevated in the BPA-alone group, whereas superoxide dismutase (SOD), myeloperoxidase (MPO), and malondialdehyde (MDA) levels did not significantly change. Transcriptional analyses revealed upregulation of the antioxidant genes Nrf2 and CAT in the PE–BPA group. Co-exposure to BPA and MPs also altered synaptic markers, including decreased brain-derived neurotrophic factor (BDNF) and Sert along with increased Nr2A expression, while inflammatory gene expression remained unaffected. **Conclusions**: These findings indicate that acute co-exposure to BPA and PE microplastics elicits early antioxidant activation and selective synaptic-related transcriptional changes in the PFC, suggesting that MPs may modulate BPA-associated molecular responses in the brain.

## 1. Introduction

The increasing reliance on plastics across industrial and consumer applications has intensified concerns about their consequences for both environmental integrity and human health [1,2]. Despite the introduction of recycling initiatives and waste-reduction strategies, it has been estimated that only a small fraction of plastic waste is effectively recycled, while the majority accumulates in landfills or natural ecosystems [3]. Polyethylene (PE) is among the most widely produced and used plastics globally, particularly in packaging and materials that come into direct contact with food commodities [4,5]. This extensive use substantially elevates human exposure to primary and secondary PE microplastics (MPs) and associated chemical pollutants, such as bisphenol A (BPA). Due to their hydrophobic surfaces and high surface-to-volume ratio, MPs can readily adsorb hydrophobic contaminants such as BPA through physicochemical interactions [6]. Once ingested, changes in environmental conditions within biological systems may promote the desorption and release of these contaminants in the gut, potentially altering their persistence, bioavailability, tissue distribution, and biological transport [7]. In addition to their potential interactions with environmental contaminants, MPs themselves have been reported to exert adverse neurobiological effects. Experimental studies suggest that micro- and nanoplastics may induce oxidative stress, mitochondrial dysfunction, neuroinflammatory responses, blood–brain barrier disruption, and behavioural alterations, potentially through cellular accumulation and the disruption of intracellular homeostasis [8]. These findings indicate that plastic particles may directly contribute to molecular and cellular processes associated with neurotoxicity. Consequently, understanding how BPA associated with PE MPs behaves is crucial for evaluating their combined impact on human health.

BPA is a synthetic xenoestrogen capable of mimicking or interfering with endogenous hormone signalling, exerting endocrine-disrupting, mutagenic, and carcinogenic effects [9]. Initially identified in vitro and subsequently confirmed in vivo, its estrogenic-like activity has raised significant concern among researchers and regulatory agencies that are currently banning its use (Commission Regulation (EU) 2024/3190 of January 2025) [10]. BPA can interact with multiple receptor families, including oestrogen, androgen, and peroxisome proliferator-activated receptors, triggering diverse regulatory pathways [11,12,13]. These interactions can disrupt endocrine homeostasis, promote oxidative stress and epigenetic alterations such as hypomethylation, and modify gene expression, ultimately contributing to a wide range of adverse health outcomes [14,15,16,17,18], including chronic conditions such as obesity, diabetes, and cardiovascular diseases [19,20].

Among the organs affected by BPA exposure, the brain is a major target given its role in integrating physiological and behavioural responses [21]. In particular, the prefrontal cortex (PFC) is of special interest due to its essential role in attention, cognition, impulse control, and the regulation of behaviours associated with disorders [22]. Given its prolonged postnatal maturation and its dependence on finely regulated neurotransmitter and endocrine signalling pathways, the PFC may be particularly susceptible to environmental stressors such as BPA and MPs. These functions are especially vulnerable to oxidative imbalance, endocrine disruption, and neurotransmitter dysregulation, making the PFC a relevant target for evaluating the neurobiological effects of BPA and MPs exposure [23]. Importantly, both BPA and MPs and nanoplastics (NPs) have demonstrated the capacity to cross biological barriers relevant to neurotoxicity. BPA readily crosses the intestinal barrier and can reach the central nervous system, whereas increasing evidence suggests that small plastic particles may translocate across intestinal epithelia and potentially access the brain either through blood–brain barrier passage or indirect systemic mechanisms [8]. This capacity for biological translocation provides mechanistic plausibility for investigating early molecular responses in the brain following oral exposure. Although the neurotoxicity of BPA is not yet fully understood, increasing evidence suggests that environmental exposure to BPA, including forms adsorbed onto microplastics, can disrupt normal brain development and function, leading to neuronal death, disrupted connectivity, and reduced myelination [21]. Several studies have shown that BPA can affect cortical development and PFC-related executive functions [24,25,26]. Furthermore, BPA exposure has been associated with an elevated risk of neurological disorders, including both neurovascular conditions such as stroke and neurodegenerative illnesses like Alzheimer’s and Parkinson’s diseases [21].

At the molecular level, BPA-induced neurotoxicity has been primarily associated with oxidative stress and neuroinflammatory processes [21]. BPA can interact with both nuclear and membrane-associated oestrogen receptors (ERs), as well as activate ER-independent signalling pathways in cortical neurons and glial cells. These interactions activate intracellular signalling cascades that promote the transcription of genes involved in pro-inflammatory responses (such as TNF-α) and cellular stress [21,27]. In parallel, BPA-induced mitochondrial dysfunction contributes to increased production of reactive oxygen species (ROS), thereby exacerbating oxidative stress [27]. Elevated ROS levels modulate redox-sensitive transcription factors, including nuclear factor erythroid 2 (Nrf2), which regulates antioxidant defence genes, and nuclear factor kappa B (NF-κB), which controls inflammation-related gene expression. Dysregulation of these pathways alters key antioxidant and pro-oxidant enzymes, such as superoxide dismutase (SOD), catalase (CAT), glutathione peroxidase (GPX), and myeloperoxidase (MPO) [21,27,28], and promotes oxidative damage to cellular macromolecules, particularly lipids, leading to increased malondialdehyde (MDA) levels. These alterations affect neurons, microglia, and astrocytes in the PFC and are recognised hallmarks of BPA-induced oxidative stress and neurotoxicity [28].

In addition to these molecular mechanisms, BPA exposure has been shown to directly interfere with neuronal signalling, synaptic plasticity, and neurotransmitter systems. Under normal conditions, neuronal and glial signalling in the PFC is tightly regulated, with microglia maintaining homeostasis, astrocytes modulating glutamate levels, and neurons balancing excitatory and inhibitory inputs [29,30,31]. Astrocytic activation, commonly assessed by glial fibrillary acidic protein (GFAP), is a key marker of neuroinflammatory and neurotoxic processes [32]. Synaptic plasticity is supported by neurotrophic factors such as brain-derived neurotrophic factor (BDNF) and monoaminergic neurotransmitters, which maintain dendritic spine integrity and circuit-level function [33,34]. BPA specifically disrupts key neurotrophic and neurotransmitter pathways in the PFC. BPA decreases BDNF and its receptor, weakening pro-survival and synaptic plasticity signalling, thereby reducing dendritic spine density and connectivity [34]. At the excitatory synapse, BPA alters NMDA receptor subunit composition, increasing Nr2A and Nr2B activity, which enhances calcium influx, triggers mitochondrial stress, and promotes excitotoxic neuronal death [35,36]. In parallel, BPA affects serotonergic signalling by modulating serotonin-related genes (including the serotonin transporter, Slc6a4/Sert, and receptors) and serotonin availability, impairing synaptic modulation and contributing to anxiety- and depression-like behaviours [37,38]. Thus, dysregulation of BDNF, Nr2A/Nr2B, and Sert represents a specific consequence of the broader oxidative stress and neuroinflammatory environment, linking molecular alterations to impaired PFC synaptic plasticity and circuit-level dysfunction.

Despite growing evidence of MPs and BPA neurotoxicity, the modulatory role of MPs as carriers of BPA remains poorly understood. In particular, limited information is available regarding the effects of acute co-exposure to BPA and PE-MPs on oxidative stress, neuroinflammatory pathways, and synaptic regulation within the PFC. Considering the capacity of PE microplastics to adsorb BPA and potentially alter its bioavailability, we hypothesized that PE-MPs could modify oxidative, inflammatory, and synaptic-related molecular responses in the rat PFC following acute oral exposure. To test this hypothesis, the aim of the present study was to evaluate the acute effects of orally administered BPA, PE-MPs, and BPA adsorbed onto PE-MPs on oxidative stress markers, inflammatory gene expression, and synaptic plasticity-related genes in the PFC of Wistar rats. Plasma biomarkers associated with oxidative stress and neurotoxicity, including glial fibrillary acidic protein (GFAP), were also assessed to determine whether brain alterations could be detected at the systemic level.

## 2. Materials and Methods

### 2.1. Chemicals and Materials

Bisphenol A (BPA, ≥99%) was obtained from Merck KGaA (Darmstadt, Germany). The certified reference material (CRM-PEBLK) consisting of medium-density polyethylene (PE) microplastics, as well as BPA-containing MPs (3002 ± 363 µg·g^−1^; SVO-CPUK-477) with a mean particle diameter of 99 µm, were acquired from SPEX CertiPrep (Stanmore, UK), now called ZeptoMetrix. Detailed physicochemical characterization of these particles, including morphology (SEM), particle size distribution (laser diffraction), and polymer identification by ATR-FTIR spectroscopy, has been previously reported [39]. Prior to oral administration, MP suspensions were magnetically stirred to minimize particle aggregation. For oral dosing in rats, a BPA stock solution (0.42 g·L^−1^) was prepared in corn oil (Fisher Scientific, Alcobendas, Spain) under magnetic agitation at 150 rpm. MPs were dispersed in a 4.5% (*w*/*v*) solution of carboxymethyl cellulose sodium salt (CMC, 90 kDa, Merck KGaA, Darmstadt, Germany) prepared in Milli-Q water at 22 g·L^−1^.

Gavage administration was carried out using glass syringes (2.5 and 5.0 mL; Scharlab, Sentmenat, Barcelona, Spain) equipped with a dosing cannula (76 mm length, 4 mm diameter). All glassware was cleaned prior to use by either heating at 400 °C for 5 h or by applying a multistep washing procedure consisting of rinsing with (i) Milli-Q water, (ii) a dichloromethane–hexane mixture, and (iii) acetone or ethanol.

All chemical reagents required for enzymatic assays and MDA quantification were purchased from Merck KGaA (Darmstadt, Germany). For RNA extraction, Tripure Isolation Reagent (Roche Diagnostics, Mannheim, Germany) was used, combined with molecular-biology-grade chloroform and isopropanol (Merck KGaA, Darmstadt, Germany). Purified RNA was ultimately resuspended in RNase-free water (Merck KGaA, Darmstadt, Germany). Reverse transcription was performed using TaqMan™ reagents (Fisher Scientific S.L., ThermoFisher Scientific Inc., Waltham, MA, USA), and real-time PCR analyses were conducted using the LightCycler^®^ 480 SYBR^®^ Green I Master mix (Roche Diagnostics, Mannheim, Germany).

### 2.2. Study Design

Twenty-four Wistar rats (12 males and 12 females) were housed individually under controlled environmental conditions, including a 12:12 h light–dark cycle, ad libitum access to food and water, temperature of 20 ± 2 °C, and 70% relative humidity. Eight-month-old animals were used for the in vivo exposure assays, with average body weights of 264.6 ± 8.7 g (females) and 438.7 ± 12.3 g (males). All procedures were performed following the principles of refinement and reduction to minimise animal discomfort and limit the number of animals used. Ethical approval was granted by the Bioethical Committee on Animal Experimentation of the University of the Balearic Islands (ref. CEEA 149-09-20), and all procedures complied with the European Directive 86/609/EEC and Spanish regulations (RD 53/2013) governing the use of vertebrate animals in research.

The experimental study included 24 rats. Animals were randomly distributed into four experimental groups (*n* = 6 per group; 3 males and 3 females): (1) Control (vehicle): received 1.7 mL corn oil·kg^−1^ plus 10 mL·kg^−1^ of 4.5% CMC, without BPA or MPs; (2) MPs: received PE MPs BPA-free (0.22 g·kg^−1^ body weight) suspended in 10 mL·kg^−1^ of 4.5% CMC, together with 1.7 mL corn oil·kg^−1^; (3) BPA: received 0.67 mg BPA·kg^−1^ body weight as freely dissolved BPA in 1.7 mL corn oil·kg^−1^, together with 10 mL·kg^−1^ body weight of 4.5% (*w*/*v*) CMC; 4) BPA-MPs: received 0.22 g BPA-containing MPs·kg^−1^ body weight suspended in 10 mL·kg^−1^ of 4.5% CMC, along with 1.7 mL corn oil·kg^−1^. The selected PE-MP dose (0.22 g·kg^−1^ body weight) was intended to represent an acute high-exposure scenario. According to the body surface area conversion method proposed by Reagan-Shaw et al. [40], this dose corresponds to a human equivalent dose of approximately 35 mg·kg^−1^. Dose selection was further guided by worst-case environmental exposure considerations based on the highest concentrations of microplastics reported in bottled water by Oßmann et al. [41]. The BPA dose (0.67 mg BPA·kg^−1^ body weight) was selected to represent a low acute exposure level within the range of previously reported toxicological studies in rodents, while remaining substantially below doses associated with overt toxic effects [42,43,44]. All treatments were administered as a single acute oral dose by gavage. Blood samples were collected via the vena cava at two sampling schedules: time A = 0 h, 0.5 h, 2 h, and 6 h (three rats per group) and time B = 0 h, 1 h, 4 h, and 24 h (the remaining three rats). Plasma was separated by centrifugation at 3000 rpm for 15 min and stored at −80 °C for subsequent determination of the most abundant BPA metabolite, that is, mono-β-D-glucuronide (BPAg). Approximately 100 µL of plasma was collected at each time point. Animals were euthanised by decapitation 24 h after administration. Additional blood samples were collected immediately following euthanizing. The brain was rapidly removed and the PFC was dissected, immediately frozen in liquid nitrogen, and stored at −80 °C until analysis.

To minimize bias, sample identities were coded, and investigators remained blinded to the experimental groups during biochemical and molecular analyses. Furthermore, to ensure reproducibility, all enzymatic assays, ELISA measurements and qPCR reactions were performed in duplicate.

### 2.3. Antioxidant Enzyme Activities

PFC samples were homogenized in 100 mM Tris-HCl buffer (pH 7.5) using a sample disperser (Ultra-Turrax^®^ T10 Disperser, IKA, Staufen, Germany), and subsequently centrifuged at 9000 rpm for 10 min at 4 °C. The resulting supernatants were collected and used for all biochemical determinations.

The enzymatic activities of CAT, SOD, GST, and MPO were quantified at 37 °C using a Shimadzu UV-2100 spectrophotometer (Shimadzu Corporation, Kyoto, Japan). CAT activity was determined in PFC homogenates and plasma following the method described by Aebi, which measures the decomposition rate of H_2_O_2_ at 240 nm [45]. SOD activity was assessed in PFC homogenates using a modified McCord and Fridovich assay, in which superoxide anions generated by a xanthine/xanthine oxidase system reduce cytochrome c, monitored at 550 nm [46]. GST activity was measured in PFC homogenates at 314 nm using reduced glutathione (GSH) and 1-chloro-2,4-dinitrobenzene (CDNB) as substrates [47]. MPO activity was quantified in PFC homogenates by tracking the oxidation of guaiacol at 470 nm [48]. All enzymatic activities were normalized to protein content, which was determined using the Bradford method with a commercial assay kit (Merck Life Science S.L.U., Madrid, Spain).

### 2.4. Malondialdehyde Determination

MDA levels were quantified in PFC homogenates and plasma using a colorimetric assay based on the reaction of MDA with the chromogenic reagent, n-methyl-2-phenylindole, to form a stable chromophore with maximal absorbance at 586 nm, following the manufacturer’s instructions. The absorbance was recorded using an Epoch microplate spectrophotometer (Bio-Tek, Agilent Technologies, Madrid, Spain).

### 2.5. RNA Extraction and Real-Time PCR

Total RNA was extracted from PFC using Tripure^®^ Isolation Reagent (Roche Diagnostics, Mannheim, Germany) following the manufacturer’s instructions. RNA purity was evaluated via spectrophotometry. Samples with an A260/A280 ratio within the acceptable range of 1.7 to 2.5 were selected, with values between 2.0 and 2.2 targeted as ideal for qPCR analysis. Any samples showing degradation or contamination outside this range were excluded. For cDNA synthesis, 1 µg of total RNA from each sample was reverse-transcribed using TaqMan Reverse Transcription Reagents (Life Technologies^®^, Carlsbad, CA, USA). Reverse transcription was carried out at 42 °C for 60 min, followed by enzyme inactivation at 99 °C for 5 min, in a final reaction volume of 10 µL.

Gene expression was evaluated by quantitative PCR (qPCR) using 3 µL of cDNA and LightCycler^®^ 480 SYBR^®^ Green I Master Mix (Roche Diagnostics, Mannheim, Germany). Amplification of target genes—including oxidative stress markers (CAT, Nrf2), inflammatory markers (NF-κB, TNF-α), and synaptic/neurotransmission-related markers (BDNF, Sert, Nr2A, Nr2B)—was performed with β-actin as the endogenous reference gene. qPCR reactions were run on a LightCycler^®^ 96 system (Roche Diagnostics, Mannheim, Germany) using an initial denaturation step at 95 °C for 10 min, followed by 45 cycles of amplification. To verify amplification specificity, a melt-curve analysis was performed at the end of each qPCR run, confirming the presence of a single, distinct peak for all primer pairs. In addition, β-actin crossing point (Cp) values showed minimal variation among experimental groups, supporting its use as a stable reference gene under the present experimental conditions. Primer sequences, accession numbers, amplicon sizes, and cycling parameters are provided in Table 1. All primer pairs were selected based on previous validation studies and standard manufacturer recommendations. Relative expression levels were calculated using the 2^−ΔΔCt^ method and normalized to β-actin and the control group.

### 2.6. Immunoassay

To evaluate whether neurobiological alterations induced by BPA and/or PE-MPs could be reflected at the systemic level, circulating GFAP was assessed as a peripheral biomarker of neurotoxicity. GFAP levels were determined in plasma using a specific rat ELISA kit (NeoBiotech, Seoul, Republic of Korea, Cat. No. NB-66-31085), according to the manufacturer’s instructions. The intra-assay and inter-assay coefficients of variation for this immunoassay were <8% and <10%, respectively.

### 2.7. Data Analysis

All statistical analyses were performed using the Statistical Package for Social Sciences (SPSS, version 29; IBM Corp., Chicago, IL, USA). Data distribution was evaluated with the Shapiro–Wilk test to assess normality of data, and homogeneity of variances with the Levene’s test. Group differences were assessed by one-way analysis of variance (ANOVA) when assumptions of normality and homoscedasticity were met, or Kruskal–Wallis test when parametric assumptions were violated. Pairwise comparisons were conducted using Benjamini–Hochberg’s post hoc test. Effect sizes were calculated as eta-squared (η^2^) for ANOVA models, epsilon-squared (ε^2^) for Kruskal–Wallis tests, and Cohen’s d for pairwise comparisons. Results are expressed as mean ± standard error of the mean (SEM), and differences were considered statistically significant at *p* < 0.05.

## 3. Results

**Figure 1:**
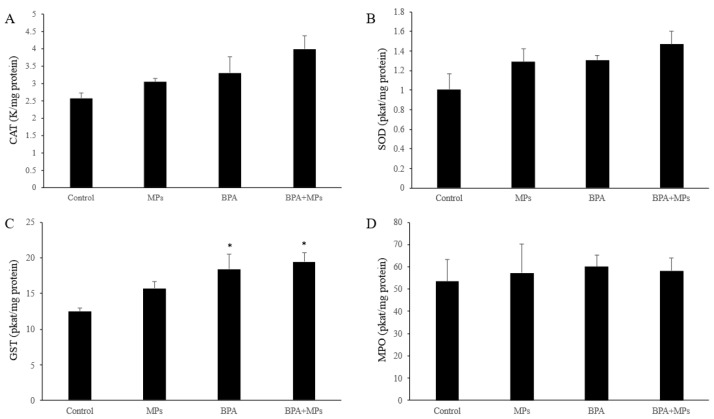
Catalase (CAT) (**A**), superoxide dismutase (SOD) (**B**), glutathione S-transferase (GST) (**C**), and myeloperoxidase (MPO) (**D**) activities in prefrontal cortex homogenates from rats either unexposed (Control) or exposed to polyethylene microplastics (MPs), bisphenol A (BPA), or their combination (BPA + MPs) (*n* = 6 per group). Results are presented as mean ± standard error (SEM). SOD and GST were analysed using one-way ANOVA (SOD: *F* (3, 20) = 2.44, *p* = 0.094, *η^2^* = 0.27; GST: *F* (3, 20) = 5.86, *p* = 0.005, *η^2^* = 0.47), whereas CAT and MPO were analysed using the Kruskal–Wallis test (CAT: *H* (3) = 10.62, *p* = 0.014, *ɛ^2^* = 0.38; MPO: *H* (3) = 0.38, *p* = 0.945). * (*p* < 0.05) indicates significant differences compared to the control group.

Analysis of oxidative stress biomarkers in the PFC (Figure 1) revealed an enzymatic response in exposed animals. Among the evaluated enzymes, CAT and GST showed the most pronounced changes. GST showed significantly increased activities in the BPA + MPs group (*p* = 0.007, Cohen’s d = 3.05) and the BPA-alone group (*p* = 0.012, Cohen’s d = 1.66) compared to the Control group. CAT activity demonstrated a significant increase in the BPA + MPs group relative to the Control (*p* = 0.007, Cohen’s d = 2.04). In contrast, exposure to MPs alone induced only mild, non-significant increases in the activity of these enzymes. The activities of SOD and MPO remained stable across groups, showing no statistically significant differences. Overall, these findings indicate a consistent modulation of antioxidant defence systems without evidence of generalized oxidative damage or enzymatic failure under the experimental conditions.

**Figure 2:**
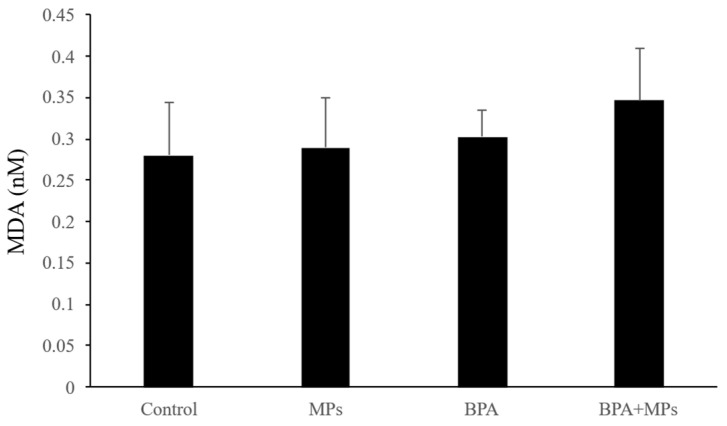
Malondialdehyde (MDA) concentration (nM) in prefrontal cortex homogenates of rats either unexposed (Control) or exposed to polyethylene microplastics (MPs), bisphenol A (BPA), or a combination of both (BPA + MPs) (*n* = 6 per group). Results are presented as mean ± standard error (SEM). Kruskal–Wallis test was used to analyse differences between groups (*H* (3) = 1.46, *p* = 0.691). No statistical differences were observed.

Despite the changes in antioxidant defences, MDA levels in PFC homogenate (Figure 2), did not differ significantly between groups, indicating no detectable differences in lipid peroxidation. This absence of changes in MDA levels was consistent across all experimental groups, supporting the lack of lipid peroxidation and reinforcing the stability of oxidative status in the PFC.

**Figure 3:**
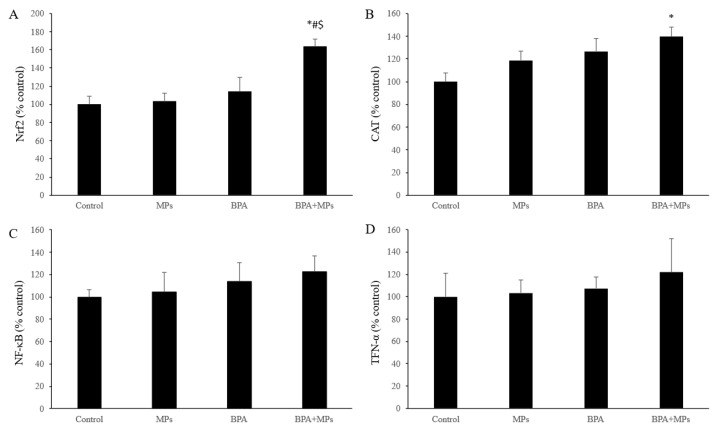
Relative expression of Nrf2 (**A**), catalase (CAT) (**B**), NF-ĸB (**C**) and TNF-α (**D**), expressed as percentage of the control group, in prefrontal cortex of rats unexposed (Control) or exposed to polyethylene microplastics (MPs), bisphenol A (BPA), or their combination (BPA + MPs) (*n* = 6 per group). Results are expressed as mean ± standard error (SEM). One-way ANOVA was used to analyse differences between groups (Nrf2: *F* (3, 20) = 7.79, *p* = 0.001, *η^2^* = 0.54; CAT: *F* (3, 20) = 3.16, *p* = 0.047, *η^2^* = 0.32; NF-ĸB: *F* (3, 20) = 0.49, *p* = 0.69, *η^2^* = 0.07; TNF-α: *F* (3, 20) = 0.23, *p* = 0.876, *η^2^* = 0.03). * (*p* < 0.05) indicates significant differences compared to the Control group. # (*p* < 0.05) indicates significant differences compared to the MPs group. $ (*p* < 0.05) indicates significant differences compared to the BPA group.

To further explore molecular responses in the PFC, the transcriptional expression of genes related to oxidative stress and inflammation was analysed (Figure 3). Nrf2 expression showed a marked upregulation in the BPA + MPs group (a 1.64-fold increase), with significant differences not only compared to the Control group (*p* = 0.002, Cohen’s d = 3.04), but also relative to the MPs-alone (*p* = 0.002, Cohen’s d = 2.94) and BPA-alone groups (*p* = 0.007, Cohen’s d = 1.65). In line with this pattern, CAT expression reached statistical significance in the BPA + MPs group compared to the Control (*p* = 0.042, Cohen’s d = 1.93, 1.39-fold increase). Notably, the increase in CAT gene expression paralleled the observed increase in CAT enzymatic activity in the BPA + MPs group, indicating a concordant transcriptional and functional response.

In contrast, no statistically significant differences were detected in the expression of the inflammatory markers NF-ĸB and TNF-α (*p* = 0.690 and *p* = 0.876, respectively). Both markers remained close to Control values across all treatment groups, indicating that acute exposure to MPs, BPA, or their combination did not elicit measurable changes in inflammatory gene expression in the PFC. These results consistently support the absence of inflammatory pathway activation in the PFC across all exposure conditions.

**Figure 4:**
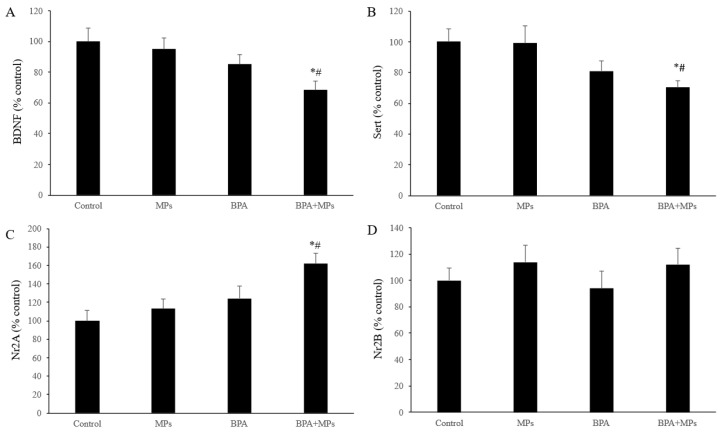
Relative expression of BDNF (**A**), Sert (**B**), Nr2A (**C**), and Nr2B (**D**), expressed as percentage of the control group, in the prefrontal cortex of rats unexposed (Control) or exposed to polyethylene microplastics (MPs), bisphenol A (BPA), or their combination (BPA + MPs) (*n* = 6 per group). Results are expressed as mean ± standard error (SEM). BDNF and Sert were analysed using the Kruskal–Wallis test (BDNF: *H* (3) = 8.97, *p* = 0.03, *ɛ^2^* = 0.28; Sert: *H* (3) = 11.43, *p* = 0.01, *ɛ^2^* = 0.38), whereas Nr2A and Nr2B were analysed using one-way ANOVA (Nr2A: *F* (3, 20) = 5.33, *p* = 0.007, *η^2^* = 0.44; Nr2B: *F* (3, 20) = 0.64, *p* = 0.597, *η^2^* = 0.09). * (*p* < 0.05) indicates significant differences compared to the control group. # (*p* < 0.05) indicates significant differences compared to the MPs group.

To evaluate potential early alterations in neurotransmission-related pathways, gene expression was subsequently analysed in the PFC (Figure 4). BDNF expression remained stable in the MPs group (*p* = 0.814), whereas the BPA + MPs group (reaching 0.68-fold of control values) showed a significant decrease compared with the Control (*p* = 0.046, Cohen’s d = 1.76) and BPA groups (*p* = 0.046, Cohen’s d = 1.11). Sert expression was significantly reduced in the BPA + MPs group (reaching 0.7-fold of control values) compared to both the control (*p* = 0.019, Cohen’s d = 1.85) and MPs groups (*p* = 0.019, Cohen’s d = 1.48), while the BPA group showed a moderate, non-significant decrease relative to Control (*p* = 0.064). For the glutamatergic markers, Nr2A expression was comparable in the Control, MPs, and BPA groups, whereas the BPA + MPs group (a 1.62-fold increase) exhibited a significant increase relative to the Control (*p* = 0.007, Cohen’s d = 2.26) and MPs groups (*p* = 0.022, Cohen’s d = 1.84). Nr2B expression remained stable across all treatment groups, with no statistically significant differences (*p* = 0.597).

Finally, we assessed biomarkers in plasma to explore peripheral responses (Table 2). In addition, pharmacokinetic analysis of BPAg was performed to confirm systemic BPA exposure and potential modulation by MPs. Plasma pharmacokinetic parameters showed a marked increase in BPAg exposure in the BPA + MPs group, with higher AUC_0_–_24_h and Cmax values compared with BPA alone (Supplementary Table S1), indicating enhanced systemic bioavailability of BPA in the presence of microplastics. No statistically significant differences were observed among groups for CAT activity, MDA levels, or GFAP concentrations. Together, these plasma biomarkers further support the absence of systemic oxidative or inflammatory alterations induced by the treatments.

## 4. Discussion

The brain, including the PFC, is a vulnerable target of BPA toxicity given its central role in cognitive processing, behavioural regulation, and synaptic plasticity. However, considerably less is known about the neurobiological consequences of simultaneous exposure to BPA and PE-derived MPs, particularly in acute exposure scenarios. In this context, our findings show that acute co-exposure to BPA and MPs elicits a mild but coordinated antioxidant response in the PFC, accompanied by early changes in synaptic-related gene expression without detectable neuroinflammation. CAT and GST activities were significantly increased in the BPA + MPs group, whereas MDA levels and SOD and MPO activities did not show significant changes. These findings indicate activation of antioxidant defences, while no differences in lipid peroxidation were detected under the experimental conditions evaluated.

Although acute co-exposure to BPA and MPs has been scarcely explored in rodent models [39], the limited available evidence from short-term exposure models in other organisms and tissues reports similar outcomes, indicating that even brief exposure may be sufficient to trigger an antioxidant response [49]. In vitro studies further support this concept, demonstrating that acute combined exposure to MPs and BPA can increase ROS production, activate antioxidant systems, and reduce cellular viability in a dose-dependent manner [50,51]. Moreover, recent reviews have highlighted that MPs and NPs may exert neurotoxic effects through oxidative stress, mitochondrial dysfunction, neuroinflammation, and disruption of intracellular homeostasis, further supporting the biological plausibility of the molecular alterations observed in the present study [8]. However, it should be considered that in vitro systems lack physiological barriers, metabolic regulation, and tissue-specific compensatory mechanisms present in vivo, which may amplify the magnitude of the observed responses compared with whole-organism models. By contrast, studies examining subacute or chronic oral exposure to BPA [52,53,54] or MPs [55,56] individually in rodents frequently report increased oxidative markers, including MDA and ROS, together with reduced antioxidant enzyme (such as CAT, SOD and GST) activity in brain tissue. Such findings suggest that the duration of exposure may influence the development of oxidative damage. In the present study, despite changes in antioxidant-related endpoints, MDA levels remained unchanged following acute exposure, indicating that lipid peroxidation was not affected at the time point evaluated. Previous studies have proposed that differences in exposure duration may contribute to distinct redox responses, with acute and chronic exposures potentially eliciting different oxidative stress profiles [57].

In addition to changes in enzymatic activity, oxidative stress responses are frequently accompanied by transcriptional adjustments in genes involved in antioxidant regulation. In line with this mechanism, both CAT and Nrf2 were significantly upregulated in the PFC of rats exposed to the combined BPA + MPs treatment. Given the central role of Nrf2 as a master regulator of antioxidant and detoxification genes, the observed upregulation may indicate the activation of redox-sensitive transcriptional pathways that coordinate cellular defence mechanisms [58]. However, Nrf2 signalling is highly context-dependent and can display transient or phase-specific activation patterns; therefore, the present increase may reflect an early or compensatory response that does not necessarily imply full restoration of redox balance [59]. Such transcriptional modulation is consistent with the enhanced antioxidant enzymatic activity recorded here and supports the interpretation of a coordinated and potentially adaptive activation of endogenous defences aimed at maintaining redox homeostasis. In this sense, previous experimental studies have reported that exposure to BPA and MPs can alter the expression of genes involved in antioxidant defence and redox homeostasis in various tissues and model organisms [60,61,62,63,64], frequently involving components of the Nrf2 signalling pathway and related antioxidant enzymes. However, reported transcriptional responses are not always consistent, as both upregulation and downregulation of Nrf2 and downstream antioxidant genes have been described depending on several experimental factors, including exposure duration, contaminant dose, and biological model [65,66]. Thus, the present results must be interpreted within the limits of an acute, single-dose design. Importantly, previous pharmacokinetic analyses performed in the same experimental animals showed a clear increase in systemic BPA exposure when co-administered with MPs, as reflected by higher BPAg AUC_0_–24 h and Cmax values in the BPA + MPs group compared with BPA alone [39] (Supplementary Table S1). Specifically, BPAg AUC_0_–24 h increased from 2325 ± 634 µg·h·L^−1^ in the BPA group to 3586 ± 526 µg·h·L^−1^ in the BPA + MPs group, while Cmax increased from 199 ± 47 µg·L^−1^ to 315 ± 50 µg·L^−1^, indicating enhanced systemic bioavailability of BPA in the presence of MPs. Thus, the lack of significant MDA elevation in either BPA-treated group is unlikely to be explained by reduced BPA absorption but rather suggests that lipid peroxidation remained effectively controlled under the acute exposure conditions evaluated. These quantitative pharmacokinetic differences provide mechanistic support for the observed biological effects and reinforce the interpretation that MPs may modulate BPA internal exposure rather than merely acting as inert co-carriers.

No significant changes were observed in the expression of the inflammatory biomarkers analysed (NF-κB and TNF-α). This absence of a measurable neuroinflammatory response is compatible with an early adaptive phase in which antioxidant defences restrain oxidative damage and prevent secondary inflammatory activation. Nevertheless, alternative explanations should also be considered, such as cell-type–specific inflammatory responses not captured by whole-tissue gene expression analysis, differences in the temporal dynamics of inflammatory signalling, or insufficient stimulus intensity to induce detectable NF-κB/TNF-α activation under the present experimental conditions [67]. In this sense, some acute studies have reported activation of inflammatory signalling after single high doses of BPA or in specific experimental contexts (e.g., pre-treatment paradigms), including increased phosphorylated NF-κB [68] or microglial activation in vitro [27]. Differences in dose, timing, tissue penetration and co-exposures likely explain these apparent discrepancies; specifically, our BPA dose and the co-administration with MPs may modulate both the kinetics and magnitude of inflammatory signalling. By contrast, chronic exposures to BPA or MPs more consistently elicit neuroinflammatory responses and increased cytokine expression (TNF-α and NF-κB), suggesting that prolonged exposure is required to engage full neuroimmune activation [54,69,70,71,72,73]. Therefore, our findings collectively support the interpretation that the acute co-exposure paradigm used here triggers early redox and synaptic transcriptional adjustments without progressing to overt neuroinflammation.

Alterations were also detected in genes involved in synaptic function and neuronal plasticity. Specifically, BDNF and Sert (SLC6A4) expression were significantly reduced in the BPA + MPs group, whereas NR2A was upregulated and NR2B remained unchanged. These findings are consistent with previous rodent studies reporting that oral exposure to BPA [74,75] and MPs [55,76,77] can disrupt BDNF/NTRK2 signalling pathways, thereby potentially impairing synaptic formation and function. Given the critical role of BDNF in neuronal survival and activity-dependent synaptic plasticity [33,34], its reduction may indicate diminished neurotrophic support and potential disturbances in synaptic maintenance in the PFC, even following acute exposure to BPA and MPs. The reduction in Sert expression observed in the BPA + MPs group further supports the presence of early disturbances in monoaminergic regulation within the PFC. The serotonin transporter plays a central role in regulating extracellular serotonin levels by mediating its reuptake from the synaptic cleft, and alterations in its expression can substantially affect serotonergic signalling [78]. Previous studies have shown that BPA exposure can alter the transcription of genes associated with the serotonergic system in the PFC, highlighting this neurotransmitter pathway as a potential target of BPA-induced adaptive neurobiological response [79]. In addition, experimental models have demonstrated that reduced Sert expression might occur in brain regions such as the PFC under conditions that disrupt serotonergic homeostasis, reflecting adaptive changes in serotonin turnover and signalling [80]. In this context, the downregulation of Sert detected in the present study may represent an early molecular response to toxicant exposure that could disturb serotonin homeostasis and contribute to alterations in synaptic plasticity. Although most previous studies have evaluated chronic exposure scenarios [74,75,76,77], our findings suggest that combined exposure to BPA and MPs may already induce detectable alterations in serotonergic pathways under acute conditions. However, because these observations are based exclusively on transcriptional endpoints, they should be interpreted as indicative of potential modulation of synaptic-related pathways rather than definitive evidence of functional synaptic dysfunction or impaired neuronal communication.

In contrast, NR2A expression was significantly increased in the BPA + MPs group. Considering the role of NR2A-containing NMDA receptors in synaptic transmission and calcium-dependent plasticity, this upregulation may reflect compensatory synaptic remodelling or a shift in excitatory signalling. A similar pattern was reported by Essawy et al., who observed increased NR2A mRNA levels in the PFC following subacute BPA exposure, accompanied by downregulation of NR2B [81]. Likewise, Khadrawy et al. suggested that alterations in NMDA receptor subunit composition may represent an adaptive response aimed at limiting excitotoxic damage induced by BPA [82]. Although the effects of MPs alone on NMDA receptor subunits remain insufficiently characterized, a recent study by Win-Shwe et al. reported decreased NMDA receptor subunit expression in rats exposed orally to high doses of PE NPs for four weeks [71]. Taken together, these findings suggest that combined exposure to BPA and MPs may disrupt glutamatergic homeostasis in the PFC, potentially contributing to alterations in excitatory balance and synaptic stability.

Plasma biomarkers were included to evaluate whether the neurobiological alterations observed in the PFC were reflected at the systemic level. No significant changes were detected in plasma oxidative stress or neuronal damage markers, such as GFAP, suggesting that the response to BPA and MPs exposure remained localized and effectively controlled in target tissues, such as the brain. While data on acute exposure is limited, our findings are consistent with the notion that short-term exposure to these contaminants may not be sufficient to elicit detectable systemic alterations [83,84], although such effects are known to depend on exposure conditions and dose. In contrast, chronic exposure studies have reported systemic oxidative stress and evidence of tissue damage reflected in circulating biomarkers [70,85]. Overall, this pattern supports a localized and early-stage response under acute exposure conditions.

Although no changes were observed in lipid peroxidation, MPO, or circulating plasma biomarkers, these negative findings should be interpreted cautiously. Rather than indicating a complete absence of biological effect, they may reflect the limited sensitivity of these endpoints and/or their detection thresholds, particularly in the context of acute exposure models, where subtle or transient alterations might not be adequately captured by these markers. In this sense, biomarkers such as MDA and MPO are often more consistently altered after repeated or prolonged exposures, when oxidative and inflammatory processes have had sufficient time to progress and accumulate [86].

Finally, the observation that combined BPA + MPs exposure elicited more pronounced molecular responses than single-contaminant treatments highlights the importance of evaluating mixture effects. MPs may act as vectors that modify BPA bioavailability or as particulate stressors that synergize with chemical toxicity, producing additive or interactive effects even in short-term exposures. Given that environmental exposures are typically to complex mixtures rather than isolated compounds, these results underscore the need for experimental models that incorporate realistic co-exposure scenarios and multiple time points to capture progression from adaptive responses to potential toxicity.

### Strengths and Limitations of the Study

One of the main strengths of this study is the investigation of the early neurobiological effects of acute oral exposure to BPA and PE-MPs. In contrast to most experimental studies based on chronic or subchronic exposure models, the present work focuses on responses occurring within 24 h after exposure, providing insight into the initial cellular adaptations that may arise when the organism first encounters these pollutants. The focus on the PFC, a brain region critically involved in cognition, behavioural regulation, and synaptic plasticity, further supports the relevance of the study. Another strength is the integrative approach used to assess different biological levels. By combining measurements of antioxidant and pro-oxidant enzymatic activities, lipid peroxidation, and the expression of genes related to oxidative stress regulation, synaptic plasticity, and inflammatory signalling, the study provides a comprehensive and holistic overview of early molecular responses in the brain. The use of well-characterized PE-MPs and controlled BPA doses also contributes to the reproducibility and environmental relevance of the findings.

However, several limitations should be considered when interpreting the results. The study included a single exposure dose and a single endpoint (24 h post-exposure), limiting the assessment of dose-dependent effects, temporal progression, and recovery processes. Therefore, the findings should be interpreted as evidence of early molecular and biochemical responses rather than a comprehensive characterization of neurotoxicity or long-term neurobiological outcomes. Future studies incorporating multiple doses, longitudinal sampling, and repeated or chronic exposure paradigms are needed to clarify the persistence and evolution of the observed effects. Additionally, although both sexes were included to improve biological representation, the number of animals per sex did not allow adequately powered sex-specific analyses or treatment × sex interaction testing. Consequently, the present results should not be interpreted as evidence of either sex-dependent or sex-independent responses, and dedicated studies will be required to address sex as a biological variable. Furthermore, although significant transcriptional alterations were identified in oxidative stress- and synaptic-related pathways, complementary protein-level and functional validation was not performed. As changes in mRNA abundance do not necessarily translate into alterations in protein expression or biological function, the observed responses should be interpreted as early molecular adaptations. Likewise, no behavioural assessments were performed, limiting the interpretation of whether the observed molecular alterations in the prefrontal cortex translate into measurable neurofunctional outcomes. In addition, cerebral accumulation or direct brain penetration of microplastics was not evaluated; therefore, the observed molecular responses cannot be definitively attributed to direct particle internalization within brain tissue. Finally, only the prefrontal cortex was evaluated. Although this region was selected because of its key role in cognition, behavioural regulation, and synaptic plasticity, other brain areas that are highly relevant to learning and memory processes, such as the hippocampus, may exhibit distinct responses to BPA and microplastic exposure and should be investigated in future studies. Taken together, forthcoming studies should incorporate protein, histological, and functional analyses together with assessments of microplastic biodistribution and tissue accumulation to determine the biological significance of these alterations and their potential impact on neuronal integrity and synaptic function and behaviour.

## 5. Conclusions

The present study suggests that acute oral co-exposure to BPA and PE-MPs can induce early molecular responses in the PFC of rats. Under the experimental conditions tested, the combined exposure triggered the coordinated activation of antioxidant defences, reflected by increased antioxidant enzyme activities and upregulation of redox-related genes, such as Nrf2 and CAT. These responses occurred in the absence of significant lipid peroxidation or detectable neuroinflammatory activation, suggesting that endogenous protective mechanisms may have contributed to maintaining redox balance during the early phase of exposure. At the same time, alterations in genes involved in synaptic regulation were observed, including decreased BDNF expression and modifications in NMDA receptor-related signalling. These findings suggest the potential modulation of molecular pathways associated with neuronal communication and plasticity; however, the functional significance of these transcriptional changes remains to be established. Notably, the combined BPA + MPs treatment generally produced more pronounced molecular responses than individual exposures, highlighting the importance of considering pollutant mixtures when evaluating early neurobiological effects of combined environmental exposures. Overall, the results suggest that acute exposure to environmental contaminants such as BPA and microplastics may induce molecular and biochemical changes indicative of early adaptive responses in the brain. Although these changes appear to reflect a compensatory stage rather than overt neurotoxicity, they may precede, but do not necessarily predict, later adverse outcomes. However, given the absence of direct structural, histological, protein-level, and functional assessments, the present findings should be interpreted primarily as evidence of early molecular and biochemical adaptations rather than definitive proof of synaptic dysfunction or neurotoxicity. Furthermore, the altered BPA tissue distribution observed following co-exposure suggests that MPs may modify BPA bioavailability and tissue exposure patterns, potentially influencing the biological responses detected in the PFC. This observation further supports the importance of evaluating toxicological outcomes within the context of combined environmental exposures, where interactions between contaminants may affect both toxicokinetics and biological responses. Future studies should further investigate these interactions by incorporating chronic exposure paradigms, longitudinal and behavioural assessments, and complementary protein level, histological, and functional validation approaches to determine whether the molecular alterations observed translate into persistent neurobiological and functional outcomes.

## Figures and Tables

**Table 1:** Primer details and conditions used in real-time PCRs.

Gene	Accession Number	Primer Sequence	Amplicon Size	Conditions
β-actin	NM_031144	Fw: 5′—AGGGAAATCGTGCGTGAC—3′ Rv: 5′—CGCTCATTGCCGATAGTC—3′	146 bp	95 °C 15 s 60 °C 30 s 72 °C 30 s
CAT	NM_012520	Fw: 5′—TGGCCTCCGAGATCTTTTCAATG—3′ Rv: 5′—GCGCTGAAGCTGTTGGGGTAGTA—3′	453 bp	95 °C 15 s 63 °C 30 s 72 °C 30 s
Nrf2	NM_057152	Fw: 5′—CTTTCGTAGCCTCCATGAAGCA—3′ Rv: 5′—AGTGTCTGGGTCATAGCATTCCA—3′	130 bp	95 °C 10 s 60 °C 30 s 72 °C 30 s
NF-ĸB	NM_199267	Fw: 5′—ACGATCTGTTTCCCCTCATCT—3′ Rv: 5′—TGCTTCTCTCCCCAGGAATA—3′	150 bp	95 °C 15 s 57 °C 30 s 72 °C 30 s
TNF-α	NM_012675	Fw: 5′—TGTCTCAGCCTCTTCTCATT—3′ Rv: 5′—AGATGATCTGAGTGTGAGGG—3′	156 bp	95 °C 15 s 55 °C 30 s 72 °C 30 s
BDNF	NM_001270630	Fw: 5′—CCAATCGAAGCTCAACCGAAGA—3′ Rv: 5′—ACTCAGGGTCCACACAAAGC—3′	349 bp	95 °C 10 s 60 °C 30 s 72 °C 30 s
Sert	NM_013034	Fw: 5′—TGCCTTTTATATCGCCTCCTAC—3′ Rv: 5′—CAGTTGCCAGTGTTCCAAGA—3′	123 bp	95 °C 10 s 60 °C 30 s 72 °C 30 s
Nr2A	NM_012573	Fw: 5′—CCGATAATCCTTTCCTCCACA—3′ Rv: 5′—TTGTAAGGGTCCGAGGGACAT—3′	76 bp	95 °C 10 s 60 °C 30 s 72 °C 30 s
Nr2B	NM_012574	Fw: 5′—ATGTCTCAGACATCTCCACGCACA—3′ Rv: 5′—TGCTGTTTCCTCCTCTTGGC—3′	76 bp	95 °C 10 s 60 °C 30 s 72 °C 30 s

**Table 2:** Plasma catalase (CAT) enzymatic activity and levels of malondialdehyde (MDA) and glial fibrillary acidic protein (GFAP) in rats unexposed (Control) or exposed to polyethylene microplastics (MPs), bisphenol A (BPA), or their combination (BPA + MPs).

	Control *n* = 6	MPs *n* = 6	BPA *n* = 6	BPA + MPs *n* = 6	*p*-Value
CAT (mk/mL)	5.56 ± 0.34	5.70 ± 0.29	6.32 ± 0.10	5.42 ± 1.00	0.271
MDA (nmol/mL)	200.3 ± 40.42	206.3 ± 34.61	217.0 ± 57.82	214.1 ± 52.99	0.966
GFAP (pg/mL)	45.87 ± 7.39	36.27 ± 4.21	32.08 ± 4.24	35.27 ± 5.04	0.647

Results are expressed as mean ± standard error (SEM). CAT and MDA were analysed using the Kruskal–Wallis test (CAT: *H* (3) = 3.91, *p* = 0.271, *ɛ^2^* = 0.04; MDA: *H* (3) = 0.27, *p* = 0.966). GFAP was analysed using one-way ANOVA (GFAP: *F* (3, 20) = 1.22, *p* = 0.307, η^2^ = 0.16).

## Data Availability

The original contributions presented in this study are included in the article. Further inquiries can be directed to the corresponding author.

## Supplementary Materials

The following supporting information can be downloaded at: https://www.mdpi.com/article/10.3390/nu18121892/s1, Table S1: Plasma pharmacokinetic parameters of bisphenol A glucuronide.

## Abbreviations

The following abbreviations are used in this manuscript:

AUC	Area under the curve
BDNF	Brain-derived neurotrophic factor
BPA	Bisphenol A
BPAg	Bisphenol A mono-β-D-glucuronide
CAT	Catalase
cDNA	Complementary DNA
CDNB	1-chloro-2,4-dinitrobenzene
Cmax	Maximum concentration
CMC	Carboxymethyl cellulose
ERs	Oestrogen receptors
GFAP	Glial fibrillary acidic protein
GPx	Glutathione peroxidase
GSH	Reduced glutathione
GST	Glutathione S-transferase
MDA	Malondialdehyde
MPO	Myeloperoxidase
MPs	Microplastics
NF-κB	Nuclear factor kappa B
NMDA	N-methyl-D-aspartate
NPs	Nanoplastics
Nrf2	Nuclear factor erythroid 2-related factor 2
Nr2A	NMDA receptor subunit 2A
Nr2B	NMDA receptor subunit 2B
NTRK2	Neurotrophic receptor tyrosine kinase 2
PE	Polyethylene
PE-MPs	Polyethylene microplastics
PFC	Prefrontal cortex
ROS	Reactive oxygen species
Sert	Serotonin transporter
Slc6a4	Solute carrier family 6 member 4
SOD	Superoxide dismutase
TNF-α	Tumour necrosis factor alpha
