# Supplementary material for: Oral Administration of Polyethylene Microplastics Induces BPA-Associated Antioxidant Activation and Synaptic-Related Transcriptional Responses in the Rat Prefrontal Cortex
Source: Nutrients. 2026 Jun 11;18(12):1892. doi: 10.3390/nu18121892 (PMC13305154; doi:10.3390/nu18121892)
Supplement: Supplementary file 1 [file nutrients-18-01892-s001.zip › nutrients-4322012-supplementary.pdf]

Oral administration of polyethylene microplastics induces BPA-associated antioxidant activation and synaptic-related transcriptional responses in the rat prefrontal cortex

Maria del Mar Ribas-Taberner [1,6], Maria Magdalena Quetglas-Llabrés [1,6,7], Llucia García-Moll [2], Manuel Jiménez-García [3,6], Joan Truyols-Vives [4], Silvia Tejada [2,3,6,7], Miguel D. Ferrer [4,5,6], Manuel Miró [2,4,6] and Antoni Sureda [1,2,6,7]

Supplementary Material

Bisphenol A (BPA, ≥99%) and the internal standard BPAg-$^{13}C_{12}$ were obtained from Merck KGaA (Darmstadt, Germany), while BPA β-D-glucuronide (BPAg) was purchased from LGC Limited (Teddington, UK). Stock solutions of BPAg (1000 mg·L^{-1}) were pre-pared in methanol and further diluted to obtain intermediate and working solutions. Matrix-matched calibration solutions of BPAg (5-100 µg·L^{-1}) were prepared by spiking blank rat plasma. Experimental details for determination of BPAg in rat plasma using ultra high-performance liquid chromatography coupled to mass spectrometry after matrix clean-up by sorptive extraction are available elsewhere [39]. Ultrapure water was obtained from a Milli-Q purification system (Millipore, Merck KGaA).

Table S1. Plasma pharmacokinetic parameters of bisphenol A glucuronide (BPAg), including area under the curve from 0 to 6 h (AUC_0–6h), area under the curve from 0 to 24 h (AUC_0–24h), and maximum concentration (Cmax), in rats unexposed (Control) or exposed to polyethylene microplastics (MPs), bisphenol A (BPA), or their combination (BPA+MPs).

		Control	MPs	BPA	BPA+MPs
BPAg	$AUC_{0\rightarrow 6h}$ (µg·h·L^{-1})	46 ± 23	30 ± 7	928 ± 276*#	1249 ± 182*#
	$AUC_{0\rightarrow 24h}$ (µg·h·L^{-1})	32 ± 11	60 ± 12	2325 ± 634*#	3586 ± 526*#
	C_{max} (µg·L^{-1})	11 ± 4	8± 2	199 ± 47*#	315 ± 50*#

Values are expressed as mean ± standard error (SEM). One-way ANOVA was used to analyse differences between groups. * ($p < 0.05$) indicates significant differences compared to the control group; # ($p < 0.05$) indicates significant differences compared to the MPs group.
